# Modeling individual development plans, mentoring support, and career preparedness relationships among Doctor of Philosophy (Ph.D.) trainees in the life sciences

**DOI:** 10.12688/f1000research.53705.2

**Published:** 2021-12-17

**Authors:** Chi-Ning Chang, Clinton A. Patterson, Nathan L. Vanderford, Teresa M. Evans

**Affiliations:** 1 Life Span Institute, University of Kansas, Lawrence, Kansas, 66045, USA; 2 Center for Teaching Excellence, Texas A&M University, College Station, Texas, 77843, USA; 3 Department of Toxicology and Cancer Biology, University of Kentucky Medical Center, Lexington, Kentucky, 40536, USA; 4 Department of Pharmacology, University of Texas Health Science Center at San Antonio, San Antonio, Texas, 78229, USA

**Keywords:** Individual Development Plan, Mentoring support, Career development, Doctoral education, Postdoctoral training

## Abstract

**Background:** As greater career development support for doctoral students and postdoctoral researchers has been emphasized, the individual development plan (IDP) has become a recommended mentoring tool. However, little is known about the effect of IDPs on mentoring and career development. This study proposed two conceptual models to examine the interrelationships among the use of IDPs, mentoring support, and career preparedness with a diverse sample of doctoral students and postdoctoral researchers in the life sciences.

**Methods:** The data leveraged for this study was collected over a three-month period, March 2016 to June 2016, as part of a cross-sectional, online survey. The survey was distributed through social media and direct email to participants enrolled in life/biological/medical or physical/applied doctoral programs at U.S. institutions. To test the proposed conceptual models, this study employed the design-based multilevel structural equation modeling.

**Results:** The analytic sample comprised 660 doctoral students and postdoctoral researchers in the life sciences from 91 institutions. The results suggested that 1) using the IDP could enhance mentoring support and career preparedness of doctoral students and postdoctoral researchers; 2) greater mentoring support and career preparedness would motivate mentees to continue utilizing the IDP with their principal investigator (PI) or advisor; and 3) females, postdoctoral researchers, and international scholars might need more support throughout the mentoring and career development process.

**Conclusions: **This research offered empirical evidence for how an IDP, mentorship, and career preparedness interact. Findings revealed the IDP enhances mentoring support and career preparedness, as well as mentoring support and career preparedness predict IDP use. We conclude the IDP is an important mentorship tool that enhances trainees’ overall career preparation.

## Introduction

At the core, doctoral education is intended as a career development catalyst. For example, doctoral students not only build their disciplinary foundation but also demonstrate capability of conceptualizing and conducting research through their dissertation. Experts agree that successful doctoral programs mentor dependent students ^1^^–^^4^ and develop them into independent scholars. ^2^^,^^5^^–^^7^ However, today is a challenging time for many doctoral students and postdoctoral researchers, regardless of discipline. Pressures such as time to degree, ^8^^,^^9^ student attrition, ^10^^,^^11^ mental health-related issues, ^12^^,^^13^ and the most recent challenges of the COVID-19 pandemic further emphasize the need for improved career development associated with the doctoral and postdoctoral environment.

Over the past decade a method used to elicit, support, and facilitate doctoral education dialogue emphasizing mentorship and career development is the individual development plan (IDP). This mentorship communication and career development tool is designed to prompt trainee development through self-reflection. Although IDP usage in non-academic sectors began in the last century when managers and organizations would align an individual’s competencies within an IDP, ^14^ the higher educational framework of the IDP was not introduced until 2002, when the Federation of American Societies for Experimental Biology ^15^ developed the IDP for postdoctoral fellows in the life sciences. The most established example of the IDP is the web-based version titled “myIDP”, which is a step-by-step platform that guides users through a process of (1) self-assessment, (2) career exploration, (3) goal-setting, and (4) implementation. ^16^

Subsequent literature has suggested how individuals in graduate education and postdoctoral training can benefit from IDP adoption to identify and develop career readiness. For example, mentees benefit from IDP usage by developing skills (i.e., technical, professional or transferable), that enhance exploration and/or awareness of career paths. ^17^^–^^19^ Researchers discovered that IDPs appear to be most effective when doctoral students and postdoctoral researchers have a positive mentoring relationship with their advisor, as well as engage in career development activities. ^20^^,^^21^ Recent IDP research indicates that 51% of surveyed doctoral students reported that the IDP was helpful to their career development. ^21^ The IDP is intended to be a vehicle doctoral students and postdocs can use to identify their career goals. ^22^ However, IDP research highlights the need to close long-standing career development infrastructure gaps within institutions and external funding solicitations that impede an individual’s career development. ^8^^,^^21^^–^^24^ For example, integrating career development experiences early and often within the doctoral curriculum, institutions can encourage an expanded career readiness within their trainees by “hiring PhD scientists to direct career development programs”. ^23^ Knowing that mentorship is closely associated with IDP effectiveness, faculty development programs are critical to improve both mentoring and the mentor’s familiarity with the IDP process. ^8^^,^^18^^–^^24^ However, Hobin and other researchers determined that only 20% of mentors were familiar with the IDP process, ^20^ and postdocs mentors often wait for their mentee to initiate career development discussions. ^20^ Similarly, additional IDP research discovered that mentees report having completed the IDP but not having discussions with their mentor. ^21^

**Figure 1.**
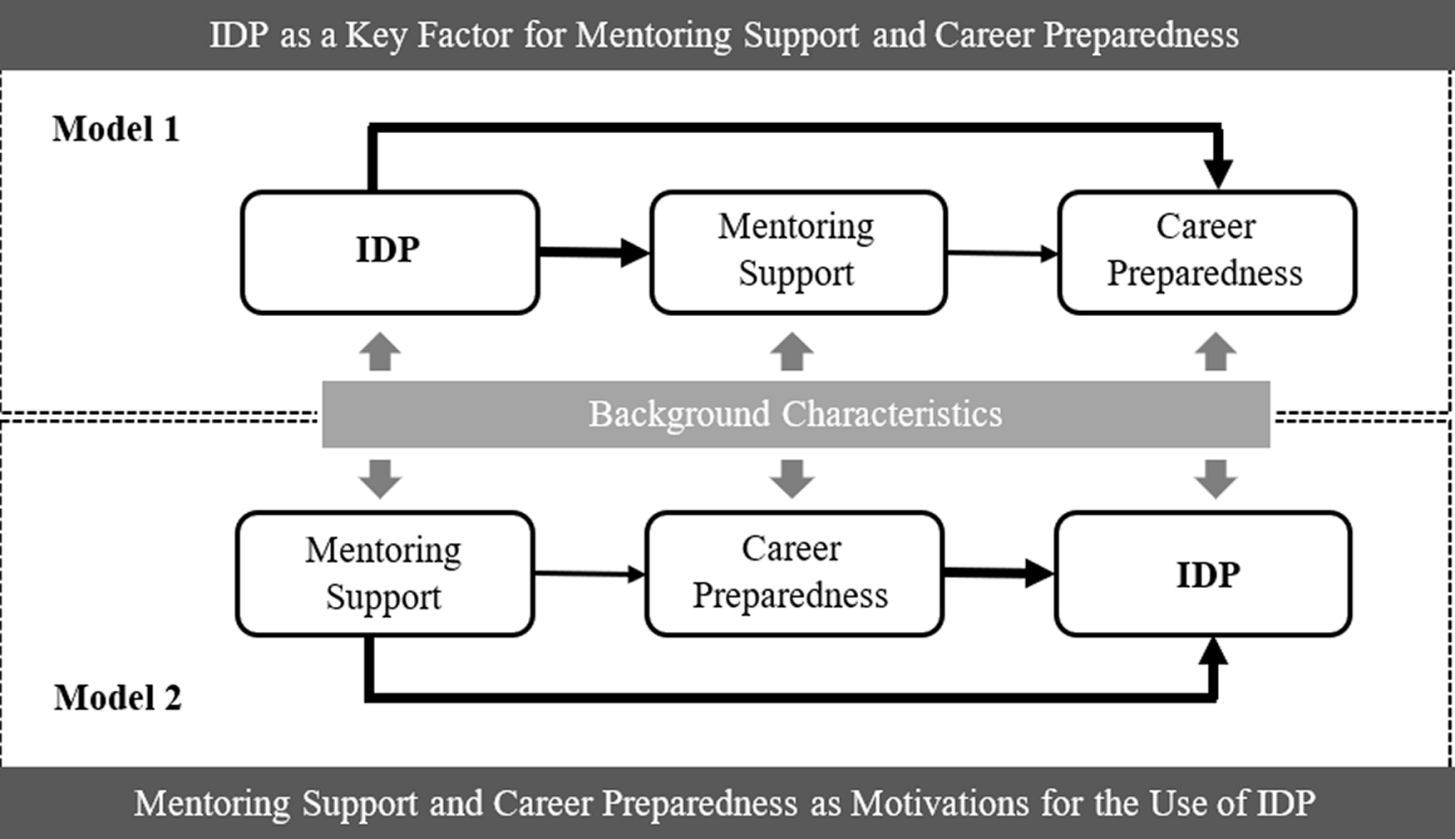
Conceptual models for the individual development plan (IDP), mentoring support, and career preparedness.

Falling under the larger recommendation to increase scholarship of mentoring, ^25^ there are calls ^19^^,^^21^^,^^22^^,^^24^ for more studies to determine the effectiveness and uniqueness of IDPs, while increasing an awareness how various trainee background and characteristics impact IDP usage and mentoring. This study further examines the impacts of the IDP by investigating the interrelationships among the IDP, mentoring support, and career preparedness for doctoral students and postdoctoral researchers. Our hypotheses are formed from existing IDP literature, ^20^^,^^21^^,^^24^ whereby two models are envisioned: 1) the IDP enhances an individual’s mentoring support and career preparedness, and 2) mentoring support and career preparedness predict the use of IDPs, as shown in Figure 1. Meanwhile, the mentee’s backgrounds are also functioning over the process.

## Methods

This research is part of a health and wellbeing study approved by the University of Kentucky (protocol 15-1080-P2H) and University of Texas Health San Antonio (protocol HSC20160025X) institutional review boards. Respondents read a cover page and consent was obtaining through the online survey web link. Survey responses were anonymous, and participants were ensured of confidentiality.

### Data/sample

The data leveraged for this study was collected as part of a cross-sectional, online survey in the spring and early summer of 2016. Survey invitations were distributed via direct email and social media (primarily Twitter and LinkedIn) to the current doctoral students and postdoctoral researchers at different United States (U.S.) institutions. A total of 864 Ph.D. trainees from 116 institutions participated in the survey. After excluding those respondents who were not in Life/Biological/Medical Sciences, the final analytic sample consisted of 660 doctoral students and postdoctoral researchers from 91 institutions.

### Measures

Mentoring support and career preparedness were collected using the five-point Likert scale from strongly disagree to strongly agree. Mentoring Support (MS) was constructed by four items, such as: “MS-1. My Principal Investigator (PI)/advisor provides real mentorship”, “MS-2. My PI/advisor is an asset to my academic and professional career”, “MS-3. My PI/advisor provides ample support”, and “MS-4. My PI/advisor positively impacts my emotional or mental wellbeing”. Career Preparedness (CP) was measured by four items including: “CP-1. I am on track to complete my training”, “CP-2. I am well prepared for completing my training”, “CP-3. I am confident about my career prospects”, and “CP-4. I am prepared for my post-training career”. The Cronbach’s alphas for MS and CP are .864 and .905, respectively. In addition to these two primary measures, we also asked participants to report the use of the IDP (i.e., whether or not they completed an IDP annually with their PI/advisor). Other individual demographic information was collected and treated as covariates, such as gender, doctoral student/postdoctoral trainee, race/ethnicity, and citizenship status. The survey questionnaire and data set are freely accessible online. ^26^^,^^27^

### Analytic strategy

To test the proposed conceptual models shown in Figure 1, this study employed the design-based multilevel structural equation models ^28^^,^^29^ by using Mplus 8.6. ^30^ This approach allows us to test the interrelationships among the variables simultaneously, handle the measurement error issue, and correct the underestimated standard errors due to the nested data structure (students clustered with institutions). Other open-source software like the *lavaan.survey* package in R could be also used to conduct the same analysis. ^31^

The models not only highlight the primary variables (IDP, mentoring support, and career preparedness), but also consider that the individual background characteristics are functioning over the process. In the analysis, for each primary variable, we also controlled for gender (female = 1; male = 0), doctoral student/postdoctoral trainee (postdoctoral trainee = 1; doctoral student = 0), race/ethnicity (Black/Hispanic/Native Americans = 1; the rest of racial groups = 0), and citizenship status (international scholar =1; citizen or permanent resident = 0). The final Mplus code and the data set we used for the analysis can be accessed at an online data repository. ^32^

## Results

### Sample characteristics and descriptive results

Among our sample (N = 660), 22% were postdoctoral researchers, 74% were female, 10% were underrepresented minorities (i.e., Black/Hispanic/Native Americans), 10% were international students/researchers, and 39 % reported they completed an IDP annually with their PI/advisor. The descriptive results for all measures and demographic information are shown in Table 1.

**Table 1.** Descriptive results for all measures.

Variables	Mean	SD	Min	Max
**IDP**	0.39	0.49	0.00	1.00
**Mentoring Support (MS)**				
▪ MS-1. My PI/advisor provides real mentorship	3.63	1.29	1.00	5.00
▪ MS-2. My PI/advisor is an asset to my academic and professional career	4.00	1.09	1.00	5.00
▪ MS-3. My PI/advisor provides ample support	3.60	1.22	1.00	5.00
▪ MS-4. My PI/advisor positively impacts my emotional or mental wellbeing	3.24	1.22	1.00	5.00
**Career Preparedness (CP)**				
▪ CP-1. I am on track to complete my training	3.88	0.98	1.00	5.00
▪ CP-2. I am well prepared for completing my training	3.55	0.99	1.00	5.00
▪ CP-3. I am confident about my career prospects	3.04	1.16	1.00	5.00
▪ CP-4. I am prepared for my post-training career	3.11	1.05	1.00	5.00
**Background Characteristics**				
▪ Female	0.74	0.44	0.00	1.00
▪ Postdoctoral Fellow	0.22	0.41	0.00	1.00
▪ Black/Hispanic/Native Americans	0.10	0.30	0.00	1.00
▪ International Scholars	0.10	0.30	0.00	1.00

*Note.* N = 660, SD = Standard Deviation, Min = Minimum, Max = Maximum, IDP = Individual development plan.

In Table 2, we describe the statistical differences of the IDP use, mentoring support, and career preparedness by trainees’ backgrounds. For example, postdoctoral fellows were less likely to use the IDP ( *β* = −.36, *p* < .01) and received lower mentoring support ( *β* = −.57, *p* < .001) compared with doctoral students. International scholars were also less likely to use the IDP ( *β* = −.24, *p* < .001) than scholars who were citizens or permanent residents of the United States.

**Table 2.** Standardized mean differences by background characteristics.

Background characteristics	IDP	Mentoring Support	Career Preparedness
**Female**			
(vs. male)	.02	−.01	−.13
*SE*	(.08)	(.10)	(.09)
**Postdoctoral Fellow**			
(vs. doctoral students)	−.36 **	−.24 *	−.27
*SE*	(.11)	(.10)	(.14)
**Black/Hispanic/Native Americans**			
(vs. other racial groups)	.07	−.03	.18
*SE*	(.17)	(.14)	(.15)
**International Scholars**			
(vs. citizen or permanent resident)	−.57 ***	.01	−.26
*SE*	(.14)	(.14)	(.14)

*Note.* N = 660, SE = Standard Error. IDP is an observed categorical variable. Mentoring support is a latent factor measured by four items (MS-1 to MS-4) shown in Table 1. Career preparedness is a latent factor measured by four items (CP-1 to CP-4) shown in Table 1. The difference test for each variable (i.e., IDP, mentoring support, and career preparedness) was examined by each background characteristic at a time.

* *p* < .05.

** *p* < .01.

*** *p* < .001.

### IDP as a key factor for mentoring support and career preparedness

**Figure 2.**
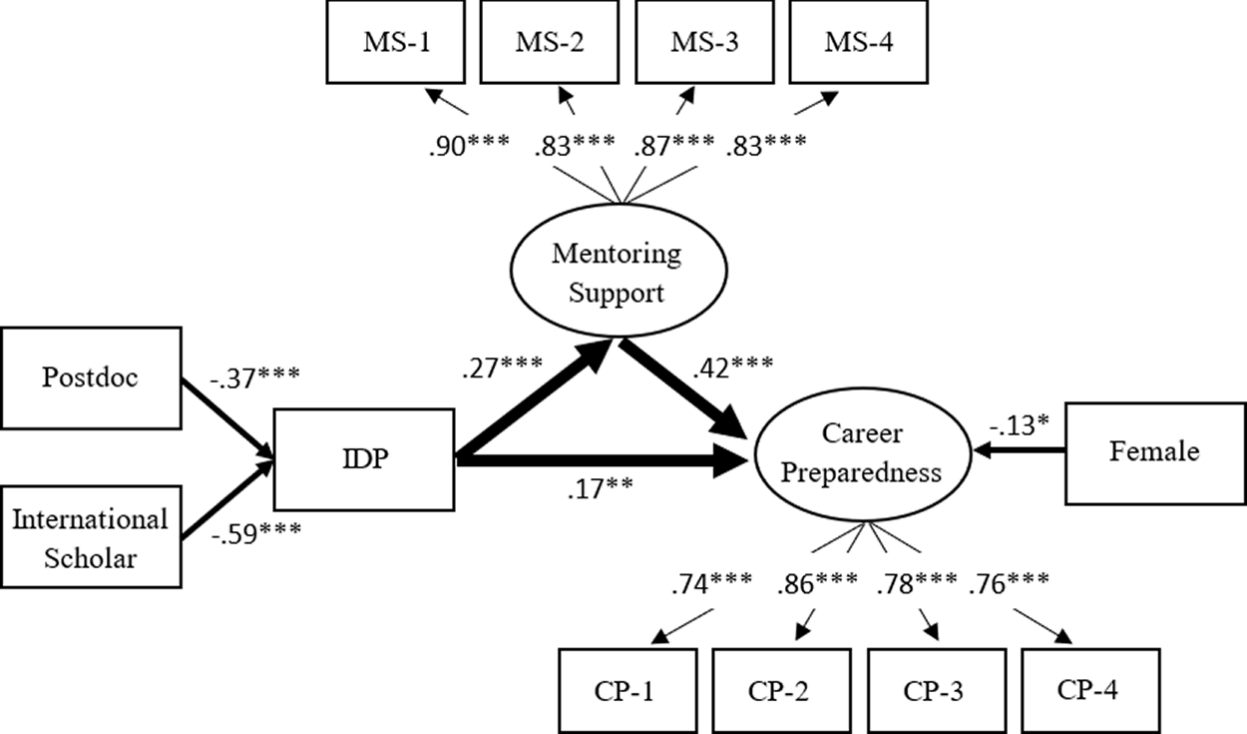
Model 1: Individual development plan (IDP) as a key factor for mentoring support and career preparedness. *Note.* Value is standardized path coefficient. The values on the light arrows are standardized factor loadings. Given that the use of the IDP is binary, the coefficients on the path from variables to IDP are standardized probit coefficients. The Primary variables (IDP, mentoring support, and career preparedness) are controlled by gender, doctoral student/postdoctoral fellow, race/ethnicity, and citizenship status. Only the statistically significant paths are shown in the figure. The reference group of the IDP is the trainee who did not use the IDP with their PI/advisor annually. The reference group of females is male. The reference group of Postdoctoral fellows is doctoral student. The reference group of international scholars is citizen or permanent resident. Oval represents a latent factor (measured by a set of indicators). Rectangle stands for an observed variable. MS-1 to MS-4 are the observed indicators of mentoring support, while CP-1 to CP-4 are the observed indicators of career preparedness. The full description for each of these indicators is shown in Table 1. * *p* < .05 ** *p* < .01 *** *p* < .001.

The statistical results indicate that model 1 adequately fits the empirical data: Root Mean Square Error of Approximation (RMSEA) = .034; Comparative Fit Index (CFI) = .965; Standardized Root Mean Squared Residual (SRMR) = .031. As shown in Figure 2, the standardize factor loadings in the model are all greater than .70, indicating good measurement validity for constructing the latent factors (mentoring support and career preparedness).

Controlling for the individual background characteristics, the results in Figure 2 reveal that the IDP shows positive effects on mentoring support ( *β* = .27, *p* < .001) and career preparedness ( *β* = .17, *p* < .01); at the same time, mentoring support is also a mediator between the IDP and career preparedness. These results suggest the use of an IDP could enhance a mentee’s career preparedness through mentoring support. However, we find that postdoctoral researchers ( *β* = −.37, *p* < .001) and international scholars ( *β* = −.59, *p* < .001) were less likely to use the IDP annually with their PI/advisor. Females had lower career preparedness ( *β* = −.13, *p* < .05) than males, but there are no significant differences among racial groups in the use of IDP, mentoring support, and career preparedness.

Overall, the use of the IDP, mentoring support, and background characteristics can account for 26% of the variance in career preparedness. The use of the IDP and background characteristics can explain 8% of the variance in mentoring support. The background characteristics can account for 5% of the variance in the use of the IDP. The unexplained variances imply that there are other unknown contributing factors also relating to these three primary variables.

### Mentoring support and career preparedness as motivations for the use of IDP

**Figure 3.**
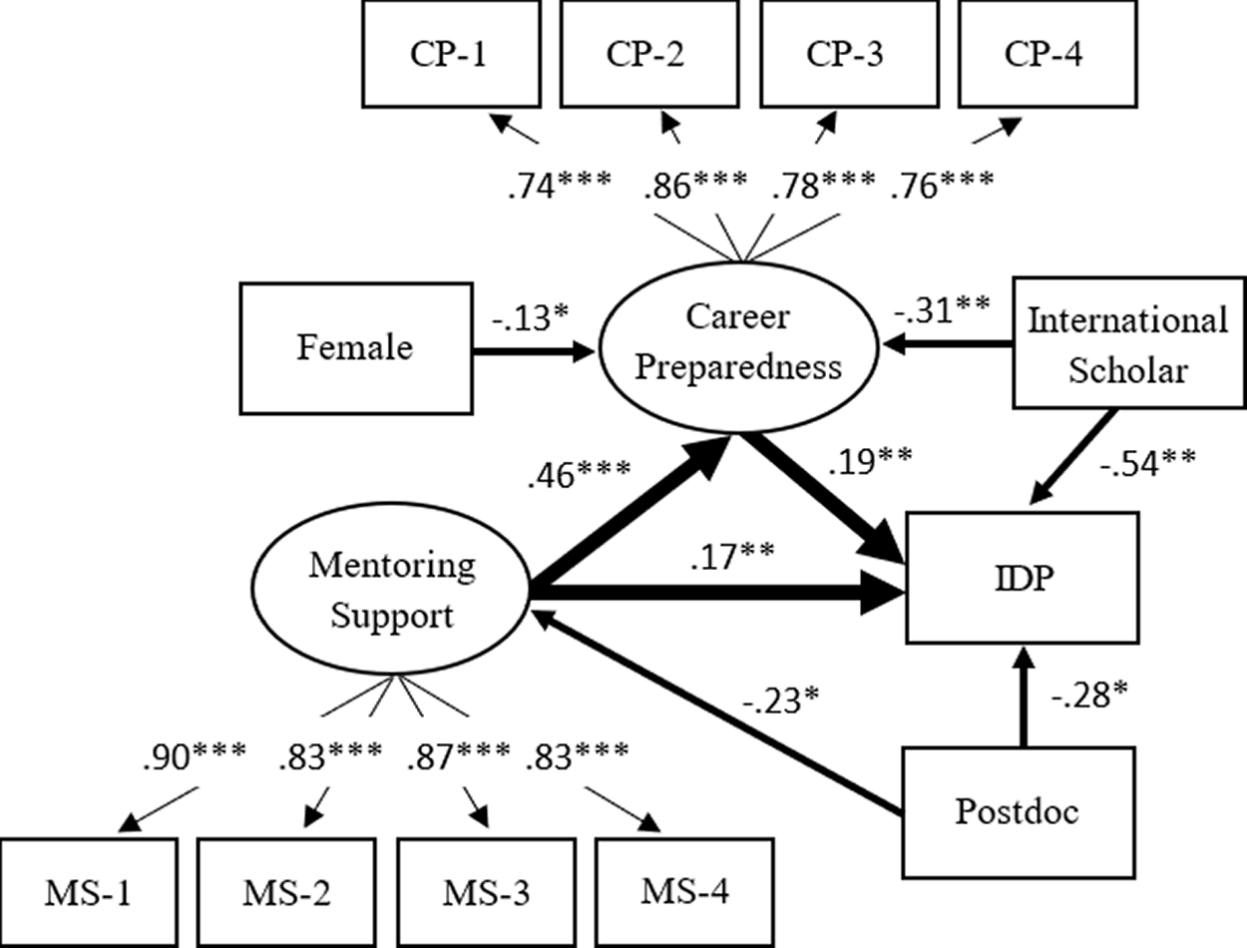
Model 2: Mentoring support and career preparedness as motivations for the use of individual development plan (IDP). *Note.* Value is standardized path coefficient. The values on the light arrows are standardized factor loadings. Given that the use of the IDP is binary, the coefficients on the path from variables to IDP are standardized probit coefficients. The Primary variables (IDP, mentoring support, and career preparedness) are controlled by gender, doctoral student/postdoctoral fellow, race/ethnicity, and citizenship status. Only the statistically significant paths are shown in the figure. The reference group of the IDP is the trainee who did not use the IDP with their PI/advisor annually. The reference group of females is male. The reference group of Postdoctoral fellows is doctoral student. The reference group of international scholars is citizen or permanent resident. Oval represents a latent factor (measured by a set of indicators). Rectangle stands for an observed variable. MS-1 to MS-4 are the observed indicators of mentoring support, while CP-1 to CP-4 are the observed indicators of career preparedness. The full description for each of these indicators is shown in Table 1. * *p* < .05 ** *p* < .01 *** *p* < .001.

The statistical results indicate that model 2 also adequately fits the empirical data (RMSEA = .034; CFI = .965; SRMR = .031). In Figure 3, the standardized factor loadings in the model also show good measurement validity for constructing the latent factors (mentoring support and career preparedness).

Over and above the background characteristics, the results in Figure 3 indicate that mentoring support has a positive effect on career preparedness ( *β* = .46, *p* < .001), while both mentoring support ( *β* = .17, *p* < .01) and career preparedness ( *β* = .19, *p* < .01) positively predict the use of the IDP. Multiple significant paths from background characteristics to three primary variables provide additional warnings. We found that the lower mentoring support ( *β* = −.23, *p* < .05) for postdoctoral researchers might partially explain why they were less likely to use the IDP ( *β* = −.28, *p* < .05). For international scholars, their lower career preparedness ( *β* = −.31, *p* < .01) indirectly revealed why they used the IDP less than U.S. citizens or permanent residents ( *β* = −.54, *p* < .01).

Overall, mentoring support, career preparedness, and individual background characteristics can explain 15% of the variance in the use of the IDP. The mentoring support and individual background characteristics can account for 24% of the variance in career preparedness. The individual background characteristics can explain only 1% of the variance in mentoring support. The unexplained variances mean that there are other unknown factors, which were not included and collected by this study.

## Discussion

As greater career development support for doctoral students and postdoctoral researchers has been emphasized, the IDP has become a commonly used mentoring tool in science, technology, engineering, and mathematics (STEM) fields. Although this tool is encouraged, its effect on mentoring support and career development is still understudied. To fill the gaps, this study investigated 660 doctoral students and postdoctoral researchers in the life sciences to test the two conceptual models by using the design-based multilevel structural equation models. The empirical evidence supports the two proposed conceptual models and connects the relationships among the use of the IDP, mentoring support, and career preparedness.

In the first model, we found that using the IDP can enhance mentoring support and career preparedness; meaning, greater mentoring support and higher level of career preparedness. Our findings affirm the IDP in practice, joining other IDP research ^20^^,^^21^^,^^24^^,^^33^ that encourage mentees to utilize the IDP to self-assess current skills and create a strategic plan with their mentors. Mentor and mentee continually prioritize and revisit the IDP to track progress and refine objectives, whereby mentees eventually achieve their career goals with mentoring support. In the second model, our finding aligned with previous research ^20^^,^^21^^,^^24^ and extends the evidence that greater mentoring support and career preparedness are associated with the use of the IDP. Although the reasons remain unknown and possibly complex, our result is similar to Hobin’s research finding that career development discussions between mentors and postdocs are often absent or lacking, ^20^ or ‘underutilized’. ^33^ Given that an IDP can establish a long-term mentorship, it is not surprising that mentees will continually hold IDP discussions with their PI or mentor when they perceive a need for mentoring support and get closer to their career goals. Recent IDP research provides evidence for enabling a customized or flexible IDP implementation process that promotes a learner-centered approach and aligns with current higher education aspirations. ^33^

This study also examined how individual background characteristics were functioning over the process. Although our results provide evidence for how the IDP and mentorship can encourage career preparedness, female trainees showed a lower career preparedness than males. Females have historically experienced notable and significant STEM challenges, such as negative stereotypes, hostile environments, and developing trainee identity. ^34^^–^^36^ Recently, mentorship was discovered to predict high levels of gender-STEM identity for women. ^35^ Aligned with an ever-increasing (and needed) higher education movement at National Institute of Health (e.g. BEST, Common Fund, T32) and National Sciences Foundation (e.g., NRT, Louis Stokes, AGEP), we recommend that institutions and faculty members should pay more attention to female scholars. Specifically, we suggest continued reduction of the STEM barriers and negative stereotypes for women, and expanded mentorship to underrepresented students in career preparedness skill development that enables a student’s transition into wider range of STEM-related careers. ^17^^,^^25^^,^^33^^,^^37^ Additionally, the results reveal that international scholars and postdoctoral researchers were less likely to use the IDP. The lower career preparedness for international scholars and the lower mentoring support for postdoctoral researchers might explain why these trainee populations used the IDP less than their counterparts. We encourage their PIs or mentors to use some well-established IDP platforms (e.g., myIDP) to identify their career goals and create action plans every year. We also recommend that mentors provide a safe and welcoming atmosphere where career preparedness discussions are the norm, not the expectation, even if that means more faculty mentoring development is needed. ^20^^,^^21^^,^^25^

While our findings show important implications for the IDP research, there are still several limitations to this study. First, the data collection (summer 2016) may be considered dated. However, our research goal was to examine the effect of the IDP. We believe it is still acceptable to use the empirical evidence to test our conceptual models. Second, even though we identified the interrelationships among the use of the IDP, mentoring support, career preparedness, and individual background characteristics, there might be other unknown factors omitted from this study, such as the different types of IDP tools, the quality of IDP discussion, etc., which could be investigated in the future studies. Third, the present study was cross-sectional and not able to properly infer the longitudinal effect of the use of the IDP. Finally, although our sample is not nationally representative, our survey sample included a diverse group of doctoral students and postdoctoral researchers in the life/biological/medical fields from 91 institutions.

Despite these limitations, this study makes methodological and practical contributions to the literature on IDP and extends the scholarship of mentoring. ^25^ First, it is one of first empirical studies to propose conceptual models for IDP research and examines the interrelationships among the IDP, mentoring support, and career preparedness with a diverse sample of doctoral students and postdoctoral researchers. Second, instead of using descriptive results to indicate the disparities of individual backgrounds in each of primary variables, this study showed how the background characteristics are functioning over the process. Third, and importantly, the findings provide the graduate and postdoctoral education community with empirical evidence and implications for the use of the IDP, as well as the need to improve mentor training. ^8^^,^^19^^–^^21^^,^^24^ Incorporating these IDP models and evidence as part of doctoral, postdoc, and early career faculty orientation would promote high quality mentoring relationships from the beginning. In short, our study suggests that using the IDP could provide career development support for both doctoral trainees and postdoctoral researchers in the life sciences.

Implementation of IDPs to improve doctoral education, postdoctoral training, and faculty mentoring will produce diversity and flexibility through meaningful and transformative educational experiences for each trainee. Especially in the current COVID-19 context, these factors will be of vast importance for the future of our research and training enterprise. This research demonstrated the empirical evidence an IDP has within mentorship and career preparedness and offered further implications the IDP is an important career development tool that enhances trainees overall career preparation.

## Data availability

### Underlying data

Figshare: Modeling individual development plans, mentoring support, and career preparedness relationships among Ph.D. trainees in the life sciences. https://doi.org/10.6084/m9.figshare.14893116.v1. ^26^

Data are available under the terms of the Creative Commons Attribution 4.0 International license (CC-BY 4.0).

### Extended data

Figshare: Survey Questions - Modeling individual development plans, mentoring support, and career preparedness relationships among Ph.D. trainees in the life sciences. https://doi.org/10.6084/m9.figshare.14893182.v1. ^27^

This project contains the following extended data: the questionnaire for this study.

Data are available under the terms of the Creative Commons Attribution 4.0 International license (CC-BY 4.0).

### Software availability

Our analysis was performed by using the statistical software, Mplus 8.6. Archived Mplus code and data set at time of publication: https://doi.org/10.5281/zenodo.5055803. ^32^

These files are available under the terms of the Creative Commons Attribution 4.0 International license (CC-BY 4.0).
