# Supplementary material for: Modeling individual development plans, mentoring support, and career preparedness relationships among Doctor of Philosophy (Ph.D.) trainees in the life sciences
Source: F1000Res. 2021 Dec 17;10:626. Originally published 2021 Jul 21. [Version 2] doi: 10.12688/f1000research.53705.2 (PMC8758970; doi:10.12688/f1000research.53705.2)
Supplement: Peer review history

# Peer review: Reviewer response for version 2

**DOI:** 10.5256/f1000research.80782.r115833

**Published:** 2022-01-13

**Authors:** Jessica Kathleen Polka

**Affiliations:** 1 ASAPbio, San Francisco, CA, USA

Thank you for helpful responses. I have no further concerns.

Is the work clearly and accurately presented and does it cite the current literature?

Yes

If applicable, is the statistical analysis and its interpretation appropriate?

I cannot comment. A qualified statistician is required.

Are all the source data underlying the results available to ensure full reproducibility?

Yes

Is the study design appropriate and is the work technically sound?

Partly

Are the conclusions drawn adequately supported by the results?

Partly

Are sufficient details of methods and analysis provided to allow replication by others?

Yes

Reviewer Expertise:

Open science, scholarly communication, early career researchers

I confirm that I have read this submission and believe that I have an appropriate level of expertise to confirm that it is of an acceptable scientific standard.

# Peer review: Reviewer response for version 2

**DOI:** 10.5256/f1000research.80782.r115832

**Published:** 2021-12-20

**Authors:** Adriana Bankston

**Affiliations:** 1 University of California Office of the President, Washington, DC, USA

No additional comments.

Is the work clearly and accurately presented and does it cite the current literature?

Yes

If applicable, is the statistical analysis and its interpretation appropriate?

Yes

Are all the source data underlying the results available to ensure full reproducibility?

Yes

Is the study design appropriate and is the work technically sound?

Yes

Are the conclusions drawn adequately supported by the results?

Yes

Are sufficient details of methods and analysis provided to allow replication by others?

Yes

Reviewer Expertise:

Higher education policy

I confirm that I have read this submission and believe that I have an appropriate level of expertise to confirm that it is of an acceptable scientific standard.

# Peer review: Reviewer response for version 1

**DOI:** 10.5256/f1000research.57120.r90082

**Published:** 2021-08-11

**Authors:** Jessica Kathleen Polka

**Affiliations:** 1 ASAPbio, San Francisco, CA, USA

**Note that I don't have adequate expertise to evaluate the structural equation modeling in this paper, hence my provisional approval.**

 In "Modeling individual development plans, mentoring support, and career preparedness relationships among Doctor of Philosophy (Ph.D.) trainees in the life sciences," Chang *et al.* analyze data from a survey of doctoral students. The design of the survey appears sound, and the resulting data enable the authors to draw valuable conclusions about IDP usage, its relationship to demographic variables, and its correlation with mentoring support and career preparedness.

 My most substantial questions surround the use of structural equation modeling, which I lack the expertise to adequately assess. I believe this work should be reviewed by an expert in that area. Nevertheless, a few issues arose for me: 

1. The statistical results are identical for both models. Does this mean that one model cannot be favored over the other, and thus, that the causal relationships can be described as a positive feedback loop, with IDP usage supporting career preparedness & mentoring support, and vice versa? Is it then possible to represent such a circular relationship in a new model, and would such a model fit the data better?
2. Why are arrows between career preparedness & mentoring support not bidirectional in both cases?  For example, imagine the trainee has good career preparedness from other sources (peers, society, internet resources, institution, etc) and has therefore selected a mentor on the basis of their support, or is therefore proactively seeking mentorship.

I also have a few more minor points:

1. In the "Conclusions" section of the abstract, I'm not understanding the first part of the sentence, which states that evidence is demonstrated but does not seem to specify the conclusion.
2. In Methods (Data/sample) - "Survey invitations were distributed via direct email to randomly chosen United States (U.S.) institutions with graduate-level programs," where were these email addresses found? Do these represent emails of graduate program coordinators, or students themselves, as implied in the following sentence?
3. In Results, the sentence beginning "With regard to individual background" could perhaps be more logically presented in a separate section about the survey responses independent of the two models.
4. Throughout the text, proofreading could help to improve readability.

Is the work clearly and accurately presented and does it cite the current literature?

Yes

If applicable, is the statistical analysis and its interpretation appropriate?

I cannot comment. A qualified statistician is required.

Are all the source data underlying the results available to ensure full reproducibility?

Yes

Is the study design appropriate and is the work technically sound?

Partly

Are the conclusions drawn adequately supported by the results?

Partly

Are sufficient details of methods and analysis provided to allow replication by others?

Yes

Reviewer Expertise:

Open science, scholarly communication, early career researchers

I confirm that I have read this submission and believe that I have an appropriate level of expertise to confirm that it is of an acceptable scientific standard, however I have significant reservations, as outlined above.

# Response

**Published:** 2021-12-13

**Authors:** Clint Patterson

Thank you for the feedback and review of our study. We appreciate your perspective, questions, and recommendations.

 We appreciate the reviewer’s question about the model fit issue. For the first question, in SEM, changing the direction of one or more paths in a model will yield an equivalent model. Equivalent models will result in precisely the same model fit. That is why our two models showed the identical model fit results (RMSEA = .034; CFI = .965; SRMR = .031). Therefore, we are not able to evaluate if one model was favored over the other, which was also not the purpose of this study. Our goal was to test if 1) the IDP enhances an individual’s mentoring support and career preparedness, and 2) mentoring support and career preparedness predict the use of IDPs. Each of the results showed the positive relationships among these three focal variables with a good model fit. For the second question, we could test a new model with a circular relationship, but the model fit will be the same given that we just change the direction of paths in the model. Therefore, we think our original models already show a good model fit to answer our research questions.

 We appreciate reviewer’s question and comments about the relationship between mentoring support and career preparedness. In this study, we considered mentoring support is one of the inputs for career preparedness (output), which was aligned with the mentoring literature (i.e., process-oriented model of mentoring; Eby et al., 2013). That was why we tested the path from mentoring support to career preparedness in both models theoretically. In practice, we agree that it is possible that trainees with good career preparedness proactively seek mentorship. To address the reviewer’s concern, we conducted additional tests for models 1 and 2 by correlating mentoring support and career preparedness (bidirectional). In updated model 1, IDP showed significantly positive effects on mentoring support (β = .27, p < .001 vs. β = .27, p < .001) and career preparedness (β = .28, p < .001), while mentoring support and career preparedness were positively correlated with each other (β = .43, p < .001). In updated model 2, both mentoring support (β = .15, p < .01) and career preparedness (β = .28, p < .01) positively predicted the use of the IDP, while mentoring support and career preparedness were positively correlated with each other (β = .38, p < .001). The updated results were still very similar and consistent with our original findings, so we decided to keep our original models and results as is in the paper.

 Thank you for the minor point about the “Conclusions” section of the abstract, which is now revised -- “This research offered empirical evidence for how an IDP, mentorship, and career preparedness interact. Findings revealed the IDP enhances mentoring support and career preparedness, as well as mentoring support and career preparedness predict IDP use. We conclude the IDP is an important mentorship tool that enhances trainees’ overall career preparation.”

 We appreciate your point about the Methods (Data/sample). The data was collected using a sample of convenience. We included institutions that made emails publicly available on their websites. These emails could have included trainees and/or program leadership including program directors or coordinators. The study was promoted on Twitter and LinkedIn, and posts were shared widely by social media users.

 Thank you for the feedback in the Results section. We ran an additional analysis to test the differences of each variable by background characteristics. This revised manuscript now includes an expanded “Sample characteristics and descriptive results” section and new Table 2 is included in this revised manuscript.

 Thank you again for your article review and methodological questions. We sincerely appreciated the feedback and incorporated into what we believe is a stronger revision.

# Peer review: Reviewer response for version 1

**DOI:** 10.5256/f1000research.57120.r90084

**Published:** 2021-08-02

**Authors:** Adriana Bankston

**Affiliations:** 1 University of California Office of the President, Washington, DC, USA

This study provides a robust characterization of the relationship between the IDP, mentoring support, and career preparedness for trainees, which is necessary for supporting graduate students and postdocs in universities. I agree with the characterization that, while this study is from 2016, the data are relevant for comparing these models and the study is valuable for the community to be aware of, and the conclusions are appropriate. The sample size and number of institutions examined also make this a robust study with important results for higher education.

 The observation that the IDP is most effective when mentoring is positive and when trainees can engage in career development activities is an interesting one, which also extends to questions related to how mentoring relationships could be improved and what systemic changes need to occur in order for career development activities to be more accepted and included into the training of graduate students and postdoctoral researchers as important components to prepare them for skill building and future jobs. For example, a recent study from PLOS Biology (Brandt *et al., *2021) showed that involvement in career development activities for PhD students is not detrimental to their research productivity.

 To this end, it was disappointing but informative to see that only 20% of mentors were familiar with the IDP process, and that in many cases mentees completed them but did not discuss them with mentors. To an extent, I agree that the mentee needs to take charge of their professional development and make sure to complete the IDP and take the initiative to set up meetings with their mentor to discuss results. But this also points to the fact that we need to incentivize a higher number of mentors to learn about the IDP, think about how institutions can assist with this goal, and whether there are positive model institutions where this occurs regularly to be used as a model for others. I’m not sure what is currently being mandated by institutions across the country in terms of the IDP. A good discussion point in terms of future directions would be to talk about incentives for mentors to use the IDP for supporting their mentees in their career development and whether institutions can put in place a reward system for those that show consistently positive results in order to improve the enterprise as a whole.

 Indeed, the report suggests that only 39% reported that they completed an IDP annually with their mentor, which isn’t a very high number considering the number of graduate students and postdocs we are training in our universities. Moreover, it seems that postdocs and international trainees aren’t using the IDP as much, which is also unfortunate to see. One question for discussion would be to think about what kind of support for career development graduate students need, which may be different from those of postdocs, and how the IDP may potentially be adapted to better suit these needs in the future. In addition, we know that international researchers typically receive less career support, and I would be curious to explore this more as to whether they use the IDP less due to language barriers or other factors that may make them feel uncomfortable to discuss career options with their mentors. Given the importance of international trainees for advancing research forward and the high percentage of the research workforce that is foreign-born with U.S. laboratories, it would be important to address how we can support them more in the discussion section.

 In terms of the survey results, I was wondering if there were any negative answers given on mentoring (or if that was an answer option) and whether trainees felt that lack of mentoring led to them feeling less prepared for a particular career path. This type of analysis could be a useful impetus for discussing systemic changes that need to occur to better support mentees, both from the perspective of the mentors and the institutions. This question could be a follow-up study to look at systemic issues and solutions to address them.

 I appreciated the remarks made in relation to IDP and long-term mentorship, as I think it’s useful to think about how often mentees approach their mentors about career goals and making sure that these conversations occur at regular intervals throughout their training. It would be useful to do a study on how often meetings between mentors and mentees related to professional and career development typically occur in universities. Given that career goals can change, I would argue this needs to occur more frequently than once a year. A longitudinal study of career development discussions that occur at various timepoints during the training of a graduate student or postdoc could be very informative.

 One question I had is whether the study results represent only STEM graduate students and postdocs, or whether they are specific to life sciences (which is more a subset of STEM). I would suggest clarifying this information in the methods section. I would also like to know more about the list of disciplines that were analyzed in the survey (if beyond STEM), and whether humanities and social sciences trainees responded differently to the survey questions as compared to those in STEM. The latter could provide useful insights into the effectiveness of the IDP.

 I would have also liked to see a breakdown between data at particular institutions, in terms of whether these mentoring relationships and consequent IDP discussions are better or worse at R1 institutions versus those that are less research intensive, such as MSIs and HBCUs for example.

 It may be interesting to survey the same population again now, in 2021 to assess pandemic impacts on the factors examined in this publication. The pandemic is likely to alter the dynamics between mentors and mentees. I would be curious to know if a higher number of trainees are completing the IDP since they are working from home using their computers. Also, if they are less able to perform bench science under the current circumstances, has this led to trainees focusing more on their career development during the pandemic and what lessons can we learn from this?

 I would like to see more of discussion on diversity aspects of this study and how different ethnic groups used and/or benefitted from the IDP (for example BIPOC, LGBTQ etc. if these options were included), as well as how these variables intersect. For example, was there a difference between the career support that female graduate students received versus female postdocs, knowing these needs may be different? What about women of color at both career stages? It would be important to discuss more details about how this kind of career advice occurs and develop some recommendations for institutions to implement programs that may address these issues for particular populations.

 Finally, I thought the models proposed were interesting and thought provoking, as well as important for explaining this complicated dynamic. But I would consider a third model, where institutions also play a role, because institutional policies may dictate how some of these interactions take place. For example, the model could be Institutional support -> mentor support -> IDP -> career preparedness, or Institutional support -> mentor support/IDP as parallel tracks -> career preparedness. In this case, mentoring and IDP could both help with career development, where mentoring either encourages the IDP or could be parallel to it, but both are dictated by institutional policies. In a systemic sense, I think it’s worthwhile considering how institutions can better incentivize mentoring and career development. Future surveys could include questions on how institutions should help graduate students and postdocs with career preparedness in addition to individual mentors, as well as how we can better prepare mentors to provide the necessary advice and guidance to their trainees.

Is the work clearly and accurately presented and does it cite the current literature?

Yes

If applicable, is the statistical analysis and its interpretation appropriate?

Yes

Are all the source data underlying the results available to ensure full reproducibility?

Yes

Is the study design appropriate and is the work technically sound?

Yes

Are the conclusions drawn adequately supported by the results?

Yes

Are sufficient details of methods and analysis provided to allow replication by others?

Yes

Reviewer Expertise:

Higher education policy

I confirm that I have read this submission and believe that I have an appropriate level of expertise to confirm that it is of an acceptable scientific standard.

## References

[1] : A cross-institutional analysis of the effects of broadening trainee professional development on research productivity. *PLoS Biol* .2021;19(7) : 10.1371/journal.pbio.3000956 e3000956 10.1371/journal.pbio.3000956 34264929PMC8282014

# Response

**Published:** 2021-12-13

**Authors:** Clint Patterson

Thank you for the review. We do agree with all the comments and wish to communicate your critique has been helpful as we revised the article. We are noting:

 Much of your feedback points to an excellent framework for future mentorship research, which we believe is important for future directions of mentoring. For example, you identified good discussion points about incentives for mentors to use the IDP and how institutions reward or value IDPs in mentoring relationships. Additionally, negative answers given to mentoring, either as a response option or open-ended response, were not included in the collected data. However, we agree with your recommendation that this mentoring relationship theme poses an important follow-up or future line of inquiry. We do agree a follow-up study that investigates institutional systemic mentoring issues and solutions to address these issues is important to high quality mentoring relationships. Your discussion point about how the COVID-19 pandemic is impacting IDP usage, research productivity, and career development is important. We believe such research could offer valuable perspectives for mentorship and the ever-evolving future workforce needs.

 The IDP is approached as a living document, and customizable in many ways, including what the mentee identifies as salient topic(s) at that moment, and which topic(s) they wish to share with their mentor(s). As such, identifying the career development needs of graduate students can be emergent and unique to the individual. We do note career development coursework is a growing and effective trend in graduate education ( source ).

 In our data sample section, we stated we removed the participant responses from a non-life sciences perspective. Therefore, our sample is fully associated with life sciences. We agree that broadening this research to include STEM and non-STEM (i.e., humanities, social sciences) would offer valuable insights into IDP implementation and effectiveness. We reviewed the collected data and determined most institutions were not MSI and/or HBCU. Unfortunately, we do not have a large and diverse enough sample to appropriately answer if IDP usage differs at institutions (i.e., R1s, MSIs, HBCUs) but agree this would be an intriguing line of future research and findings could offer specific institutional implications for mentorship. Additionally, our study’s variable was the biological gender. We recommend future studies that investigate how the IDP is incorporated and/or benefits marginalized populations (BIPOC, LBGTQ, etc.).

 Lastly, your suggestion to make the institutional role more explicit with the models make sense; however, we do not have the data to support this recommendation. However, the idea aligns with two emerging notions in literature and is of interest for our authorship team. First, *The* *Science of Effective Mentoring in STEMM *(2019) reports institutions certainly influence the mentoring environment at their school, and even offered examples (including an IDP) for formalizing mentorship within and across the institution. Institutional approaches to mentoring are not only lines of inquiry, but also an implementation area, for some of our authorship team members. For example, the recent Transformative Doctoral Education Model (TDEM) explicitly recognizes the institution (including programs, mentors, and doctoral student) as an internal driver for the doctoral ecosystem.

 Again, our thanks to the reviewer for their thoughtful feedback and insightful recommendations for this study and future IDP research considerations.
